# Flaxseed Oil Alleviates Trimethyltin-Induced Cell Injury and Inhibits the Pro-Inflammatory Activation of Astrocytes in the Hippocampus of Female Rats

**DOI:** 10.3390/cells13141184

**Published:** 2024-07-11

**Authors:** Nataša Mitrović, Marija Adžić Bukvić, Marina Zarić Kontić, Milorad Dragić, Snježana Petrović, Marija Paunović, Vesna Vučić, Ivana Grković

**Affiliations:** 1 Department of Molecular Biology and Endocrinology, Vinča Institute of Nuclear Sciences, National Institute of the Republic of Serbia, University of Belgrade, 11000 Belgrade, Serbia; marinazaric@vin.bg.ac.rs (M.Z.K.); istanojevic@vin.bg.ac.rs (I.G.); 2 Department for General Physiology and Biophysics, Faculty of Biology, University of Belgrade, 11000 Belgrade, Serbia; amarija@bio.bg.ac.rs (M.A.B.); milorad.dragic@bio.bg.ac.rs (M.D.); 3 Center for Laser Microscopy, Faculty of Biology, University of Belgrade, 11000 Belgrade, Serbia; 4 Group for Nutritional Biochemistry and Dietology, Centre of Research Excellence in Nutrition and Metabolism, Institute for Medical Research, National Institute of Republic of Serbia, University of Belgrade, 11000 Belgrade, Serbia; snjezana.petrovic@imi.bg.ac.rs (S.P.); marija.paunovic@imi.bg.ac.rs (M.P.); vesna.vucic@imi.bg.ac.rs (V.V.)

**Keywords:** hippocampus, trimethyltin, flaxseed oil, alpha-linolenic acid, neuroinflammation, astrocytes, neuroprotection

## Abstract

Exposure to the neurotoxin trimethyltin (TMT) selectively induces hippocampal neuronal injury and astrocyte activation accompanied with resultant neuroinflammation, which causes severe behavioral, cognitive, and memory impairment. A large body of evidence suggests that flaxseed oil (FSO), as one of the richest sources of essential omega-3 fatty acids, i.e., α-linolenic acids (ALA), displays neuroprotective properties. Here, we report the preventive effects of dietary FSO treatment in a rat model of TMT intoxication. The administration of FSO (1 mL/kg, orally) before and over the course of TMT intoxication (a single dose, 8 mg/kg, i.p.) reduced hippocampal cell death, prevented the activation of astrocytes, and inhibited their polarization toward a pro-inflammatory/neurotoxic phenotype. The underlying protective mechanism was delineated through the selective upregulation of BDNF and PI3K/Akt and the suppression of ERK activation in the hippocampus. Pretreatment with FSO reduced cell death and efficiently suppressed the expression of inflammatory molecules. These beneficial effects were accompanied by an increased intrahippocampal content of n-3 fatty acids. In vitro, ALA pretreatment prevented the TMT-induced polarization of cultured astrocytes towards the pro-inflammatory spectrum. Together, these findings support the beneficial neuroprotective properties of FSO/ALA against TMT-induced neurodegeneration and accompanied inflammation and hint at a promising preventive use of FSO in hippocampal degeneration and dysfunction.

## 1. Introduction

Neurodegenerative diseases represent a broad spectrum of disorders that differ in their clinical presentation and symptoms but often share common pathological features: the progressive damage of neurons leading to neurological, cognitive, and behavioral deficits [1]. Pathological processes can have a variety of causes, including genetic and environmental factors [2,3]. The organotin compound trimethyltin (TMT), which is widely used in industry and agriculture, is highly neurotoxic [4]. It causes severe behavioral, cognitive, and memory impairment; aggressiveness; ataxia; sensory neuropathy; and seizures in both humans and laboratory animals [5,6,7]. TMT specifically targets the limbic system, in particular the hippocampal formation, and causes neuronal loss and behavioral disturbances similar to Alzheimer’s disease (AD) [6,7,8]. In addition, TMT-induced neurodegeneration shares pathological mechanisms with many neurodegenerative diseases, such as the alteration of glutamate transmission, intracellular calcium overload, oxidative stress, mitochondrial dysfunction, neuroinflammation, and cell death [5,8].

In the rat hippocampus, the degeneration of pyramidal neurons shows a delayed onset (two to four days after intoxication), becomes prominent at the end of the first week after TMT intoxication, and becomes severe after 3 weeks [5,9,10]. TMT triggers an immediate reactive response in astrocytes in vitro [11,12] and in vivo [9,10,13], and it affects neuronal survival in a Ca^2+^-dependent manner [14]. An early change in astrocyte morphology preceding neuronal loss, particularly reactive astrocyte phenotypes, and their dynamic remodeling after TMT intoxication in rats has been described [10]. TMT polarizes astrocytes towards the pro-inflammatory phenotype, characterized by the robust expression of neurotoxic complement C3 (C3) and inflammatory mediators such as interleukin (IL)-1β, IL-10, IL-6, and tumor necrosis factor-α (TNF-α), both in vitro and in vivo [9,12,15,16]. Given that pro-inflammatory astrocytes may contribute to a variety of neurodegenerative diseases, they represent a valuable target for the treatment of central nervous system (CNS) disorders.

Due to their broad spectrum of pharmacological and biological activities, natural products could be promising alternatives for the prevention/amelioration of neuroinflammation associated with neurodegenerative diseases. Flax (*Linum usitatissimum* L., FS) is cultivated worldwide as a remarkable functional food. Flaxseed oil (FSO) contains the highest amounts of plant-derived essential omega-6 (ω-6/n-6) and omega-3 (ω-3/n-3) polyunsaturated fatty acids (PUFAs) such as linoleic acid (LA, C18:2) and α-linolenic acid (ALA, C18:3), respectively, but also lignans, high quality proteins, soluble fiber, phenolic compounds, and vitamin E [17]. Numerous data support the benefits of FSO in the diet for a range of cardiovascular diseases, hypertension, diabetes, dyslipidemia, inflammation, cancer, gastrointestinal health, and hormonal status, as well as in brain development and function [17,18,19]. A large body of evidence suggests that FSO also has neuroprotective properties. It reduces depressive-like behavior [20,21,22], ameliorates learning and memory decline in obese rodents [23], improves functional motor recovery after ischemic stroke [24], reduces neurotoxicity in both adult and developing rodent brains [25,26,27], ameliorates brain dysfunction caused by diabetes [28], etc.

Several studies suggest that ALA may be the bioactive compound responsible for the neuroprotective effects of FS/FSO [17]. The uptake of ALA per se and/or its long-chain metabolites may mediate a variety of molecular and cellular events that improve neuronal survival and restore CNS function [29,30]. ALA increases neurogenesis in conjunction with an increase in brain-derived neurotrophic factor (BDNF) protein levels, and it stimulates synaptic plasticity and synaptogenesis [31,32]. It also activates the antioxidative system, suppresses inflammatory responses to Aβ_25−35_-induced neurotoxicity in C6 glial cells [33], inhibits apoptosis following hydrogen peroxide (H_2_O_2_)-induced oxidative stress in SH-SY5Y neuronal cells [34], and protects SH-SY5Y cells against β amyloid toxicity [35].

Therefore, the current study aimed to provide the neuroprotective effects of FSO on neurodegeneration induced by the TMT neurotoxin in female rats. We investigated potential mechanisms by which FSO/ALA prevent different aspects of TMT-induced hippocampal damage, including neuronal death and the pro-inflammatory response of astrocytes in vivo and in vitro. Our results indicate that FSO/ALA alleviate TMT-induced cell death and inhibit the inflammatory activation of astrocytes in the hippocampus of female rats.

## 2. Materials and Methods

### 2.1. Animals and Treatments

One hundred and twenty adult female rats of the Wistar strain weighing 200–220 g were obtained from the local colony maintained in the animal facility of the VINČA Institute of Nuclear Sciences—National Institute of Republic of Serbia, University of Belgrade. Appropriate actions were taken to alleviate the pain and discomfort of the animals in accordance with the compliance with European Communities Council Directive (2010/63/EU) for animal experiments, and the research procedures were approved by the Ethical Committee for the Use of Laboratory Animals of VINČA Institute of Nuclear Sciences—National Institute of Republic of Serbia, University of Belgrade, Belgrade, Republic of Serbia (Application No. 323-07-02057/2017-05). Animals were housed (3–4/cage) under standard conditions: 12 h light/dark regime, constant ambient temperature (22 ± 2 °C), and free access to food and water.

**Figure 1:**
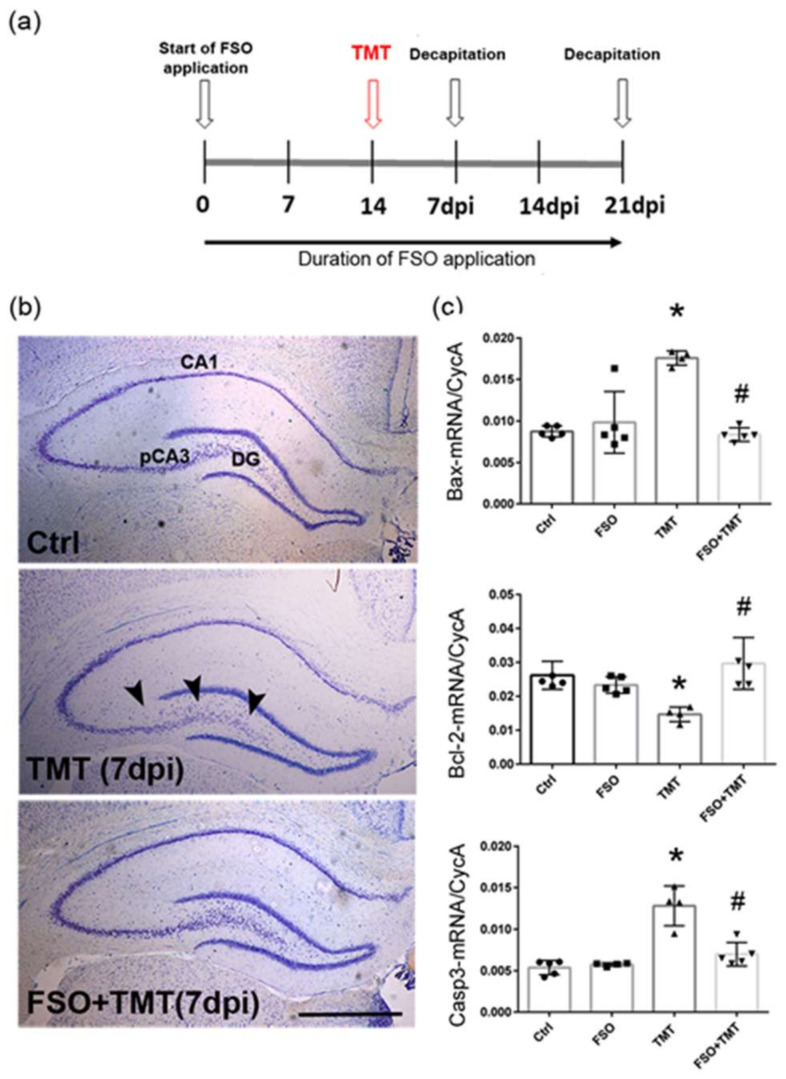
FSO prevented TMT-induced hippocampal damage 7 days post intoxication. (**a**) A schematic illustration of an experimental design. (**b**) Representative thionin-stained coronal sections obtained from control (Ctrl), trimethyltin (TMT), and flaxseed oil (FSO)-treated TMT-intoxicated (FSO+TMT) animals. Arrowheads indicate injured neuronal cell layers in the hippocampus, while the laminar organization of pCA3 subfield was regular in the FSO-treated TMT sections. Scale bar = 500 μm. (**c**) The abundance of transcript coding Bax, Bcl-2, and Caspase3 (Casp3) assessed by RT-qPCR in Ctrl, FSO, TMT, FSO+TMT animals. Bars represent mean target mRNA abundance relative to CycA (± SD) from *n* = 5 hippocampi per group. Significance shown inside the graphs: * *p* < 0.05 or less relative to Ctrl; # *p* < 0.05 or less relative to TMT. pCA3, proximal CA3 subfield of the hippocampus; DG, dentate gyrus; dpi, days post intoxication.

To evaluate the effect of FSO on TMT-induced pathology, the animals were divided into 4 groups based on whether they received FSO treatment and/or underwent TMT intoxication: a control group without any treatment (Ctrl), a group with FSO treatment (FSO), a TMT-intoxicated group without FSO treatment (TMT), and an FSO-treated TMT-intoxicated group (FSO+TMT). Females from the FSO and FSO+TMT groups were continuously treated with flaxseed oil (Granum^®^, Hajdukovo, Serbia commercial, 1 mL/kg, orally by using a syringe) for two weeks. The dose for FSO was chosen based on data in the literature showing its beneficial anti-inflammatory effects in various organ systems in both males and females [36,37,38,39,40]. All animals adapted to daily FSO administration without any difficulties. The fatty acid composition of FSO (Table 1) was confirmed by gas chromatography analysis (Shimadzu 2014, Kyoto, Japan). On day 14, a part of untreated and FSO-treated animals received a single dose of TMT (TMT and FSO+TMT groups), and the application of FSO was continued for both the FSO and FSO+TMT group. The rats were injected intraperitoneally (i.p.) with TMT (8.0 mg/kg, body weight) dissolved in 0.9% saline and then returned to their home cages [9,10]. We chose two time points after TMT intoxication since we previously described that a significant number of damaged neurons were already observed 7 days after intoxication (early stage of TMT-induced neurodegeneration) [9,10]. This is followed by the almost complete disappearance of staining in neuronal somata in the proximal and medial CA3 regions (p/mCA3) 21 days post intoxication (dpi; the late stage of TMT-induced neurodegeneration) [9]. Thus, a part of the animals (*n* = 8/group) was sacrificed 7 days post TMT intoxication and after 3 weeks of total FSO treatment. Most of the animals (*n* = 23/group) were sacrificed 21 days post TMT intoxication (TMT group) and after 5 weeks of total FSO treatment (FSO and FSO+TMT groups). A schematic illustration of the experimental design is shown in Figure 1a. All animals were decapitated by a small animal guillotine (Harvard apparatus), and brains were isolated for tissue processing. 

### 2.2. Primary Astrocyte Cultures and Treatments

Primary astrocyte cultures were prepared from 1–2-day-old male Wistar rat pups. Briefly, cortices were isolated in ice-cold phosphate-buffered saline (PBS), and the meninges were carefully and thoroughly removed to avoid contamination of the culture with fibroblasts and meningeal cells. Tissues were mechanically dissociated, and the cell suspension was centrifuged, resuspended, and passed through 21G (ø 0.8 mm) and 23G (ø 0.6 mm) needles. Cultures were seeded and maintained in growth medium (Dulbecco’s modified Eagle medium, DMEM, supplemented with 10% FBS, D-glucose to a final concentration of 25 mmol/L, 100 IU/mL penicillin, and 100 μg/mL streptomycin, all from Thermo Fisher Scientific-Gibco, Waltham, MA, USA) in a humidified atmosphere of 5% CO2/95% air at 37 °C. The culture medium was replaced 24 h after seeding and then every 2–3 days. Primary microglia and oligodendrocyte precursor cells (OPCs) were removed by vigorous shaking on a plate shaker with additional mechanical washing using a 1 mL pipette before culture reaching confluence, if needed. The astrocyte culture prepared by this protocol consisted of >98% of GFAP^+^ cells, while less than 2% of DAPI stained nuclei were associated with Iba1^+^ cells, as previously reported [41]. No other markers were detected. For specific experimental designs, the cells were seeded in 24-well plates for MTT assays, in 6-well plates for RT-qPCR, and on PLL-coated glass coverslips (ø 15 mm, PLL—Sigma-Aldrich, St. Louis, MO, USA) for immunofluorescence labelling. For in vitro treatment, alpha-linoleic acid (Sigma-Aldrich, St. Louis, MO, USA, L2376) was dissolved in ethanol to yield 1 mg/mL stock. Afterwards, stocks of serial ALA concentrations were prepared in DMEM so that the highest final ethanol amount did not exceed 0.2%. The neurotoxin TMT was freshly prepared before the treatments and dissolved in water at 1 mM concentration, so the final amount of water would not affect the astrocyte viability or metabolism. Both treatments solely and combined were added to the growth medium to obtain 80–90% confluent cultures.

### 2.3. MTT Assay

The cytotoxic effects of ALA and TMT were evaluated by assessing total mitochondrial activity using the MTT assay (3-[4,5-dimethylthiazol-2-yl]-2,5-diphenyltetrazolium bromide, Sigma-Aldrich, St. Louis, MO, USA). Primary astrocytes were seeded in 24 wells (1.5 × 10^4^ cells/cm^2^). Astrocytes were treated for 24 h and, subsequently, new medium containing 10% MTT (5 mg/mL) was added to the cultures. Cells were incubated at 37 °C for 30 min, and the formazan generated by total mitochondrial activity was dissolved in DMSO. The optical density was measured at 540 nm using an Epoch elisa multiple reader. Measurements were performed on three independent astrocyte cultures and in triplicate. Formazan formation as an indirect measure of cell death was expressed as a percentage of untreated controls.

### 2.4. Histology, Immunohistochemistry, and Immunofluorescence

Brains (*n* = 5 animals/group) were carefully removed from skulls and fixed in 4% paraformaldehyde/0.1 M phosphate buffer (pH 7.4), without transcardial perfusion. After cryoprotection in graded sucrose solutions (10–30% in 0.2 M phosphate buffer) at 4 °C, 20 µm thick hippocampal coronal sections were mounted on gelatin-coated slides, air-dried for about 2 h, and stored at −20 °C until use. Sections of the dorsal hippocampus were taken starting from level Bregma 2.6 to 3.6 mm, according to the rat stereotaxic atlas of Paxinos and Watson [42].

Nissl staining, immunohistochemistry, and immunofluorescence were performed as described previously [9]. For immunohistochemistry, sections were probed with anti-rat glial fibrillary acidic protein (GFAP) antibodies (1:100 dilutions, UC Davis/NIH NeuroMab Facility 73-240, ab10672298) overnight at 4 °C. After washing in PBS, sections were incubated with goat anti-mouse HRP-conjugated secondary antibody (1:150 dilutions, R&D Systems, Minneapolis, MN, USA, HAF007). For double immunofluorescence labeling, brain sections were immunostained for mouse anti-rat GFAP antibodies (1:100 dilution, UC Davis/NIH NeuroMab Facility 73-240, ab10672298) and donkey anti-mouse Alexa Fluor 647 antibodies (1:400 dilution, Invitrogen, Waltham, MA, USA, A-31571), as well as anti-complement C3 antibody (1:100 dilution, Thermo Fisher Scientific, Waltham, MA, USA, PA1-29715) and donkey anti-goat Alexa Fluor 488 (1:400 dilution, Invitrogen, Waltham, MA, USA, A-11055). The primary and secondary antibodies were applied separately. All secondary antibodies were tested (negative controls). A controls without primary antibodies did not show staining, autofluorescence, or any unspecific binding of the secondary antibodies. Sections were mounted in Mowiol (Calbiochem, Burlington, MA, USA).

The cells on coverslips were prefixed in 4% PFA, subsequently permeabilized (0.05% Triton in PBS, for 15 min, at RT), and blocked for 1 h with 5% BSA in 0.01 M PBS. Cells on coverslips were incubated overnight at +4 °C with mouse anti-GFAP antibody (1:500; Sigma Aldrich, G3893), rabbit anti-iNOS antibody (1:50, Invitrogen PA1-036), or goat anti-C3 antibody (1:300, Invitrogen PA1-29715). Corresponding secondary donkey anti-mouse AF488 antibody (1:200 in PBS; Invitrogen A-21202), donkey anti-rabbit AF555 (1:200, Invitrogen A-21428), or donkey anti-goat AF647 antibody (1:200, Invitrogen A-21447) were incubated for 2 h at RT. Nuclei were counterstained with DAPI for 15 min, at RT, and cells were mounted on slides with MOWIOL solution. The omission of the primary antibodies resulted in the absence of any specific immunoreactivity (ir).

Digital images of thionin staining and immunohistochemistry were examined using a LEITZ DM RB light microscope (Leica Mikroskopie & Systems GmbH, Wetzlar, Germany), a LEICA DFC320 CCD camera (Leica Microsystems Ltd., Heerbrugg, Switzerland), and LEICA DFC Twain Software v. 7.3 (Leica Mikroskopie & Systems, Germany) under 5× magnification. Digital images of double immunofluorescence were taken on a confocal laser-scanning microscope (LSM 510, Carl Zeiss, Oberkochen, Germany), using Ar multi-line (457, 478, 488, and 514 nm) and HeNe (543 nm) lasers, under 63× magnification, equipped with an AxioCam ICm1 camera (Carl Zeiss, Germany).

### 2.5. Gene Expression Analysis by RT-qPCR

Total RNA from the whole hippocampus formations (*n* = 5 animals/group) or from astrocyte cultures in 6-well plates were extracted using TRIzol Reagent (Invitrogen, Waltham, MA, USA) according to the manufacturer’s protocol as previously described [9]. The concentration and the purity of the RNA were assessed using OD260 and the OD260/OD280 ratio, respectively. Complementary DNA (cDNA) species were synthesized using a High-Capacity cDNA Reverse Transcription Kit (Thermo Fisher Scientific, Waltham, MA, USA), as previously described [9,43]. Quantitative real-time PCR was performed using Power SYBR™ Green PCR Master Mix (Applied Biosystems, Waltham, MA, USA) and an ABI Prism 7000 Sequence Detection System (Applied Biosystems, MA, USA) as described previously [9]. The primer sequences used for amplification are given in Table 2. Relative quantification was performed using the comparative 2^−ΔΔCt^ method, using peptidylprolyl isomerase A, also known as cyclophilin A (CycA), as a reference gene. Samples obtained from 5 animals for each experimental group or each sample obtained from cell culture were run in duplicate. In each run, internal standard curves were generated by several-fold dilutions of generated cDNA to check the amplification efficacy. Melt curve analysis was performed at the end of every experiment to confirm the formation of a single PCR product.

### 2.6. Preparation of Membrane Fraction

After decapitation, hippocampi from the same experimental group (5–6 brains/group) were isolated for preparations of crude membrane fractions that contained a bulk of synaptosomes and other membrane fragments, such as glial cells and neuronal membranes, as described previously [44]. Hippocampi were suspended in 10 volumes of ice-cold medium (0.32 M sucrose, 5 mM Tris–HCl, pH 7.4) and homogenized in a Teflon/glass homogenizer (clearance 0.20 mm) at 900 rpm. A crude nuclear fraction and cell debris were removed by centrifugation at 1000× *g* for 10 min. Collected supernatants were further centrifuged at 16,000× *g* for 30 min to obtain a crude membrane fraction, which was resuspended in 5 mM Tris–HCl at pH 7.4. All steps were carried out at 4 °C. The samples were aliquoted and stored at −80 °C until use. The P2 fractions were separately isolated from 5–6 animals without pooling the tissue. The protein content was determined using bovine serum albumin as a standard.

### 2.7. SDS-PAGE and Immunoblotting

SDS-PAGE and immunoblotting were performed as previously described [44]. The membranes were incubated with the primary antibodies (Table 3) overnight at 4 °C, and following vigorous washing procedures, they were incubated with an appropriate horseradish–peroxidase-conjugated secondary antibody (1: 10000 dilutions in TBST or PBST) for 1 h at room temperature. The visualization of the specific bands was performed on an iBright CL1500 Imaging System (Thermo Fischer Scientific, Waltham, MA, USA) with a commercial chemiluminescence kit (Immobilon Western Chemiluminescent HRP substrate, Millipore, Darmstadt, Germany). A densitometric analysis was performed using the ImageJ 1.53t software package, and the optical density of each band was normalized by the optical density of the β-actin band from the same lane.

### 2.8. Hippocampal Fatty Acid Analysis

Total lipid extracts of *n* = 6 hippocampal tissues per group were prepared using chloroform/methanol solvents of various compositions and successive centrifugation, as described previously [45]. Hippocampal tissue (~150 mg) was homogenized in 4 mL of chloroform/methanol (1:2, by volume) with 10 mg/100 mL 2,6-di-tert-butyl-4-methylphenol (BHT) added as an antioxidant. The resulting mixture was centrifuged for 20 min at 3200 rpm to separate it into two layers. The supernatant was evaporated to dryness, and the residue was dissolved in 3 mL chloroform/methanol (2:1, by volume). Low-molecular-weight contaminants were eliminated via partition with 2 mL chloroform/methanol (2:1, by volume) and 1 mL 0.1 mol/L KCl. After centrifugation for 20 min at 3200 rpm, the upper layer was discarded, and the lower lipid layer was evaporated to dryness. The residue was dissolved in 3 mL chloroform/methanol (2:1, by volume). Direct transesterification of fatty acids was carried out by mixing 500 µL of the suspension with 1.5 mL 3 mol/L HCl in methanol, vortexing it for 30, and heating it to 85 °C for 45 min. After cooling to room temperature, 1 mL hexane was added, vortexed for 30 s, and centrifuged for 10 min on 1800× *g*. The hexane extract (~500 µL) containing fatty acid methyl esters was evaporated under a stream of nitrogen to complete dryness. For fatty acid analysis, a sample was dissolved in 10 µL of hexane, and 1 µL was injected in a Shimadzu GC 2014 gas chromatograph (Kyoto, Japan). The chromatograph was equipped with a flame ionization detector on an Rtx 2330 column (60 m × 0.25 mm ID, film thickness 0.2 μm, Restek, Bellefonte, PA, USA). Separation was obtained over a 51 min period with an initial temperature of 140 °C held for 5 min. The temperature was then increased to 220 °C at a rate of 3 °C/min and held at the final temperature for 20 min. The injection was performed with a split ratio of 50:1 and constant flow operating mode at 11 mL/min, with helium as carrier gas. The injector temperature was 220 °C and the detector temperature was 240 °C. The identification of individual fatty acids was performed by comparing retention times with standard mixtures (PUFA-2 and/or 37 FAMEs mix, Supelco, Bellefonte, PA, USA). The following fatty acids were analyzed: 16:0 palmitic acid, 16:1n-7 palmitoleic acid, 18:0 stearic acid, 18:1n-9 oleic acid, 18:1n-7 vaccenic acid, 18:2n-6 linoleic acid, 18:3n-6 g-linolenic acid, 18:3n-3 a-linoleic acid (ALA), 20:3n-6 dihomo-gamma-linolenic acid, 20:4n-6 arachidonic acid (AA), 20:5n-3 eicosapentaenoic acid (EPA), 22:4n-6 docosatetraenoic acid, 22:5n-3 docosapentaenoic acid (DPA), and 22:6n-3 docosahexaenoic acid (DHA). Individual fatty acids were expressed as a percentage of the total identified fatty acids.

### 2.9. Data Analysis

All values are means ± SD of *n* independent determinations, as well as separate culture preparations. Student’s *t*-test was used for two-sample comparisons at the 95% confidence level. For multiple comparisons, we used two-way ANOVA followed by Tuckey’s post hoc multiple comparison test at a significance level of *p* < 0.05 and a target power of 80–95%. Data were analyzed in the Prism—GraphPad 6.07 software package.

## 3. Results

### 3.1. FSO Attenuated TMT-Induced Cell Death

To investigate whether FSO prevents neuronal injury as early as 7 days post TMT intoxication (7 dpi), as well as at the late stage of neurodegeneration (21 dpi) [9,10], we used Nissl staining for the gross assessment of hippocampal cytoarchitecture. As in Ctrl, regular laminar organization in the p/mCA3 regions was observed in the FSO-treated TMT sections from 7 dpi, indicating that continuous FSO pretreatment protected the hippocampus from TMT-induced early neuronal injury (Figure 1b). FSO alone did not induce any significant effects compared to Ctrl; thus, the results for TMT and TMT+FSO were compared to Ctrl. It was reported that neurons died due to TMT-induced caspase-dependent apoptosis [46]. The Bax-to-Bcl-2 expression ratio is used as a crucial marker to distinguish apoptosis in cells, as well as caspase3 (Casp3), a key factor involved in initiating the apoptotic process. The prevention of apoptosis was evaluated by Bax-, Bcl-2-, and Casp3-mRNA levels at the early stage. The results of two-way ANOVA for examined components of cell death pathways are presented in Supplementary Material Table S1. The qPCR analysis showed that following 7 dpi, TMT led to the upregulation of the pro-apoptotic markers Bax-mRNA (*p* < 0.01) and Casp3-mRNA (*p* < 0.01) and the downregulation of anti-apoptotic Bcl-2-mRNA (*p* < 0.01), in respect to Ctrl, which resulted in a high Bax/Bcl-2 ratio (Figure 1c). In FSO-treated TMT-intoxicated hippocampi (FSO+TMT group), Bax- and caspase-3-mRNA levels were reverted to control levels, and they were decreased compared to TMT-intoxicated hippocampi (FSO+TMT group, *p* < 0.01, and *p* < 0.001, respectively), whereas Bcl-2-mRNA was reverted to Ctrl levels and upregulated compared to TMT animals (*p* < 0.001), indicating the neuronal protective effect of FSO at the early stage of TMT-induced neurodegeneration. All further experiments were performed at the late stage of TMT-induced neurodegeneration.

**Figure 2:**
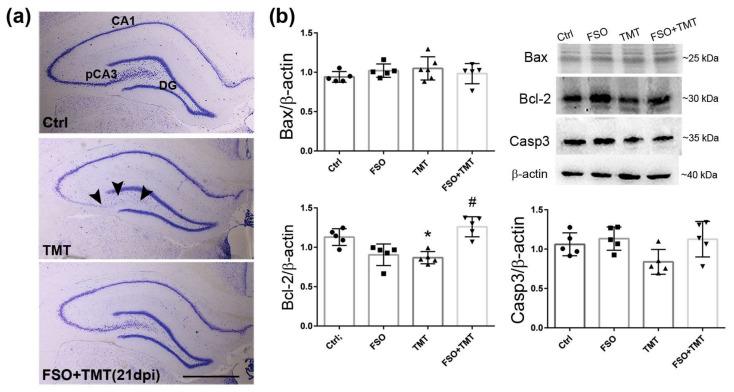
FSO pretreatment prevented TMT-induced hippocampal damage 21 days post intoxication. (**a**) Representative thionin-stained coronal sections obtained from control (Ctrl), trimethyltin (TMT), and flaxseed oil (FSO)-treated TMT-intoxicated (FSO+TMT) animals. Arrowheads indicate injured neuronal cell layers in the pCA3 subfield of the hippocampus, while the laminar organization of pCA3 was regular in the FSO-treated TMT sections. Scale bar = 500 μm. (**b**) Relative protein abundances and representative immunoblots of Bax, Bcl-2, and Caspase3 (Casp3) in Ctrl, FSO, TMT, and FSO+TMT animals. Bars represent mean protein abundance relative to β-actin ± SD, from *n* = 5 hippocampi per group. Significance shown inside the graphs: * *p* < 0.05 or less relative to Ctrl; # *p* < 0.05 or less relative to TMT. pCA3, proximal CA3 subfield of the hippocampus; DG, dentate gyrus; 21 dpi, 21 days post TMT intoxication.

As in Ctrl, Nissl staining showed regular laminar organization of p/mCA3 regions in FSO-treated TMT sections at the late stage of neurodegeneration (21 dpi) (Figure 2a), indicating that continuous FSO pretreatment protected hippocampi from TMT-induced progressive neuronal loss. FSO alone did not induce any significant effect compared to Ctrl; thus, the results for TMT and TMT+FSO were compared to Ctrl. The expression of key apoptotic molecules was also determined by Western blot analysis 21 days post TMT intoxication (Figure 2b). While there were no changes in the protein expression of Bax and caspase-3, TMT induced a significant decrease in Bcl-2 protein expression (*p* < 0.001, compared to Ctrl), while FSO counteracted this effect in the FSO+TMT group (*p* < 0.01).

### 3.2. FSO Affects Signaling Molecules Involved in Cell Survival

**Figure 3:**
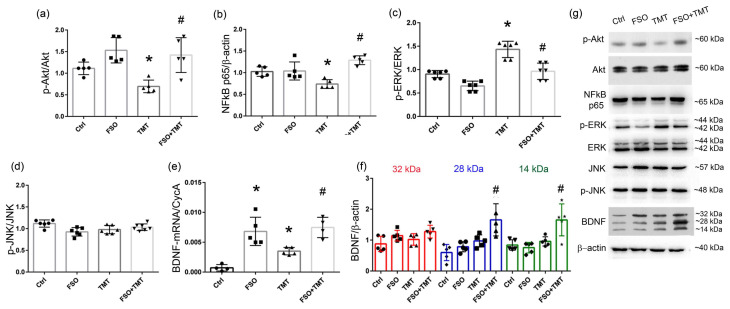
FSO affects BDNF/Akt/ERK signaling pathways 21 days post TMT intoxication. Relative protein abundances of p-Akt/Akt (**a**), NFκB p65 (**b**) p-ERK/ERK (**c**), p-JNK/JNK (**d**), and BDNF (**f**) in the crude hippocampal membrane fractions from control (Ctrl), flaxseed oil (FSO)-treated, trimethyltin (TMT)-treated, and flaxseed oil-treated TMT-intoxicated (FSO+TMT) animals assessed by Western blot analysis. Bars represent mean protein abundance relative to β-actin ± SD, from *n* = 5–6 hippocampi per group. (**g**) Representative immunoblots of investigated proteins of Ctrl, FSO, TMT, and FSO+TMT animals. (**e**) The abundance of transcript coding BDNF assessed by RT-qPCR. Bars represent mean target mRNA abundance relative to CycA (± SD) from *n* = 5 hippocampi per group. Significance shown inside the graphs: * *p* < 0.05 or less relative to Ctrl; # *p* < 0.05 or less relative to TMT.

Further, we investigated whether FSO modulates PI3K/Akt, extracellular signal-regulated kinases (ERKs), C-Jun N-terminal kinase (JNK), and nuclear factor-κB (NFκB), well-known signaling molecules involved in the regulation of cell survival. Western blot analysis revealed a significantly decreased level of Akt protein phosphorylation (p-Akt) in TMT-intoxicated hippocampi, while the p-Akt level was significantly increased in FSO-treated TMT-intoxicated animals (*p* < 0.01 compared to TMT, Figure 3a). Furthermore, we evaluated whether FSO treatment may affect the activation of NFκB, as one of the molecular players interacting with multiple upstream and downstream signaling molecules, including PI3K/Akt, as it plays a role in the regulation of expression of a variety of genes involved in the control of inflammation, cell death, and cell survival [47]. In TMT-intoxicated hippocampi, the level of the NFκB p65 subunit was significantly decreased (*p* < 0.01) in relation to Ctrl, while the protein level of the p65 subunit was increased in FSO-treated TMT-intoxicated animals (*p* < 0.001 compared to TMT, Figure 3b). FSO alone induced a significant decrease in p-ERK levels (*p* < 0.05), while TMT increased them (*p* < 0.001, Figure 3c) compared to Ctrl. In FSO-treated TMT-intoxicated animals, p-ERK levels were significantly decreased (*p* < 0.001 compared to TMT).

Further, Western blotting was performed to examine whether FSO affects the activation of JNK, a key MAPK family member that crucially regulates the apoptosis of nerve cells [48]. Post hoc analyses revealed no significant differences in teh phosphorylation level of JNK in both the TMT and FSO+TMT groups (Figure 3d).

It was proposed that the intake of polyunsaturated fatty acids affects BDNF, which is important for the survival, growth, and maintenance of specific types of neurons and which induces a long-lasting neurorestorative effect [32,49]. Thus, we investigated whether FSO, one of the richest sources of essential fatty acids, affects the expression of neurotrophic factor BDNF. The results of two-way ANOVA for qPCR and Western blot analysis are presented in the Supplementary Material in Table S2. At the mRNA level, significantly increased BDNF-mRNA levels (*p* < 0.001 compared to Ctrl) were observed in the FSO group, as well as in FSO-treated TMT-intoxicated hippocampi (*p* < 0.001 compared to TMT, Figure 3e). Western blotting revealed 32 kDa/pro-BDNF, 28 kDa/truncated BDNF, and 14 kDa/mature BDNF (Figure 3f). Our analysis showed that FSO treatment of TMT-intoxicated animals induced significant upregulation of 28 kDa and 14 kDa BDNF compared to TMT-intoxicated hippocampi (*p* < 0.01 and *p* < 0.001, respectively) and compared to FSO alone (*p* < 0.001 for both forms, Figure 3f).

### 3.3. FSO Alters Components of Glutamatergic Transmission

**Figure 4:**
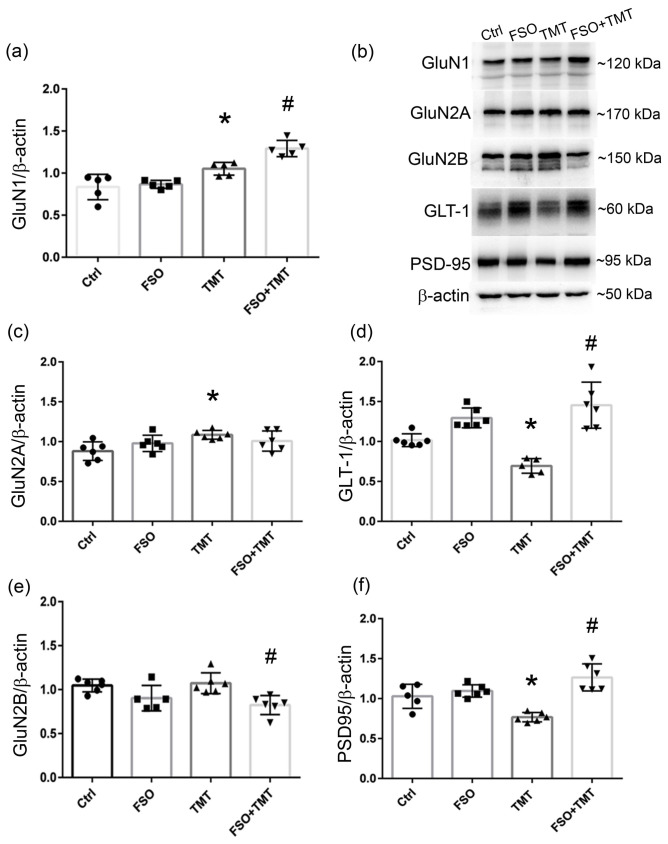
FSO affects the expression of components of glutamatergic transmission 21 days post TMT intoxication. Relative protein abundances of GluN1 (**a**), GluN2A (**c**), GLT-1 (**d**), GluN2b (**e**), and PSD-95 (**f**) in the crude hippocampal membrane fractions of control (Ctrl), flaxseed oil (FSO)-treated, trimethyltin (TMT)-treated, and flaxseed oil-treated TMT-intoxicated (FSO+TMT) animals assessed by Western blot analysis. Bars represent mean protein abundance relative to β-actin ± SD, from *n* = 5 hippocampi per group. (**b**) Representative immunoblots of investigated proteins of Ctrl, FSO, TMT, and FSO+TMT animals. β-actin was probed to ensure equal protein loading. Significance shown inside the graphs: * *p* < 0.05 or less relative to Ctrl; # *p* < 0.05 or less relative to TMT.

Since it was proposed that that diets/supplementation enriched in omega-3 fatty acids affect NMDA receptor (NMDAR)-mediated neurotransmission [30,32], which is impaired in numerous neurodegenerative disorders including TMT neurotoxicity [7], we investigated whether TMT and/or FSO affects the expression of NMDAR subunits (GluN1, GluN2A, and GluN2B), specific glutamate transporter GLT-1, and a major anchoring and scaffolding protein associated with the receptors (PSD-95). The results of two-way ANOVA are presented in the Supplementary Material, in Table S3. The protein levels of GluN1 were slightly increased in the TMT group (*p* < 0.05, compared to Ctrl) and in the FSO+TMT group in relation to both FSO groups (*p* < 0.01) (Figure 4a). Similar to GluN1, TMT intoxication led to a slight increase in the protein level of the GluNR2A subunit (*p* < 0.05, compared to Ctrl; Figure 4c). On the other hand, the protein level of GluN2B was decreased in the FSO+TMT group (*p* < 0.01, compared to TMT) and without protein alteration in the TMT group (Figure 4e). The protein level of GLT-1, the glial glutamate transporter responsible for 90% of glutamate reuptake [50], was significantly decreased in the TMT group (*p* < 0.01, compared to Ctrl), while FSO prevented this decrease in the FSO+TMT group (*p* < 0.001, compared to TMT) (Figure 4d). Together with cell death and the loss of synapses, the protein level of PSD95 was also decreased in response to TMT intoxication (*p* < 0.01, compared to Ctrl), while FSO prevented this alteration and even increased PSD95 protein abundance in the FSO+TMT group (*p* < 0.001, compared to TMT) (Figure 4f).

### 3.4. FSO Attenuated Gliosis at the Late Stage of TMT Intoxication

**Figure 5:**
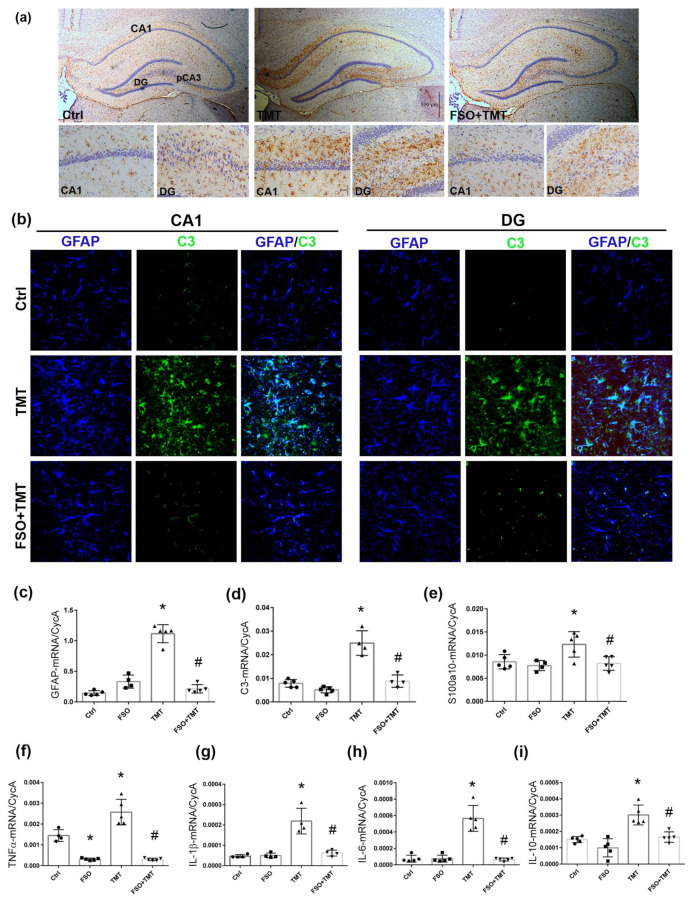
FSO prevented TMT-induced astrogliosis and neuroinflammation. (**a**) Immunohistochemical staining of GFAP in control (Ctrl), trimethyltin (TMT), and flaxseed oil (FSO)-treated TMT-intoxicated (FSO+TMT) animals. Scale bar = 500 μm. Smaller panels represent high-magnification images (40×) of GFAP-ir in CA1 and DG in Ctrl, TMT, and FSO+TMT groups. Scale bar = 50 μm. (**b**) Representative images of double immunofluorescence labeling directed to GFAP (blue) and C3 (green) in CA1 and DG subregions in Ctrl, TMT, and FSO+TMT experimental groups. C3-immunoreactivity (*ir*) was observed in the GFAP^+^-positive cells of TMT-intoxicated hippocampi, while C3-ir was not detected in the GFAP^+^ astrocytes of FSO-treated TMT-intoxicated hippocampi. Scale bar = 50 μm. The abundance of transcript coding astrocyte marker GFAP (**c**), pro-inflammatory astrocytic marker C3 (**d**), protective astrocyte marker s100a10 (**e**), and inflammatory markers TNFα (**f**), IL-1β (**g**), IL-6 (**h**), and IL-10 (**i**) assessed by RT-qPCR in Ctrl, FSO, TMT, and FSO+TMT animals. Bars represent mean target mRNA abundance relative to CycA (± SD) from *n* = 5 hippocampi per group. Significance shown inside the graphs: * *p* < 0.05 or less relative to Ctrl; # *p* < 0.05 or less relative to TMT. DG, dentate gyrus.

TMT triggers an immediate reactive response in astrocytes, which are the major source of inflammatory factors in the hippocampal tissue at the late stage of TMT-induced neurodegeneration [9,10,13,15]. Here, we evaluated whether continuous FSO treatment attenuates TMT-induced astrocyte reactivity. As shown in Figure 5a, FSO administration prevented the TMT-induced massive reactivation of GFAP-ir cells, although occasional astrocytes with enlarged cell bodies in the pCA3 subregion might be observed. FSO alone did not produce any of the effects on the level of GFAP-ir compared to Ctrl; thus, all examined groups were compared to Ctrl. In order to provide additional evidence of the effect of FSO on astrocytes following TMT intoxication, we examined the levels of GFAP-mRNA; C3-mRNA, a marker of proinflammatory/harmful astrocytes; and S100a10-mRNA, a marker of neuroprotective astrocytes. The results of two-way ANOVA are presented in the Supplementary Material, in Table S4. As we reported previously, there was significant upregulation of GFAP- and C3-mRNA levels in TMT-intoxicated hippocampi compared to Ctrl (*p* < 0.001 for both), while FSO prevented such an increase for examined mRNAs (*p* < 0.001 for both GFAP- and C3-mRNA in respect to TMT; Figure 5c,d). The level of S100a10-mRNA was also increased after TMT (*p* < 0.05, relative to Ctrl), indicating that TMT-induced reactive astrogliosis exerts a complex molecular signature with a predominantly pro-inflammatory phenotype. FSO prevented an increase in s100a10 (*p* < 0.05 in respect to TMT, Figure 5e). Furthermore, we performed double immunofluorescence labeling against GFAP and C3 (Figure 5b). As we have shown previously [9], C3-ir was observed in the GFAP^+^-positive cells of TMT-intoxicated hippocampi, while C3-ir was not detected in the GFAP^+^ astrocytes of FSO-treated TMT-intoxicated hippocampi.

Furthermore, we assessed whether FSO affects the expression of inflammatory molecules in hippocampal tissue (Figure 5f–i). The results of two-way ANOVA are presented in the Supplementary Material, in Table S4. As we shown previously, the levels of TNF-α-, IL-1β-, IL-10-, an IL-6-mRNA were significantly increased after TMT intoxication (*p* < 0.001 for all examined mRNAs compered to Ctrl), and this increase was fully prevented by the FSO treatment of the TMT-intoxicated group (*p* < 0.001 for TNFα, IL-6, and IL-1β and *p* < 0.01 for IL-10), suggesting that FSO may attenuate neuroinflammation in the hippocampus. FSO alone also decreased the mRNA level of TNFα (*p* < 0.001) compared to Ctrl.

### 3.5. FSO Alters Hippocampal Fatty Acid Composition

One of the mechanisms by which natural oils enriched in n-3 PUFAs may exert their beneficial effects and preserve tissue structure/organization is through changes in tissue fatty acid composition [51]. Thus, we investigated whether FSO protects the hippocampus by altering hippocampal fatty acid composition at the late stage following TMT intoxication. A comprehensive list of fatty acids is compiled in Table 4. The results of two-way ANOVA are presented in the Supplementary Material, in Table S5. Notable differences were observed in the hippocampal levels of palmitic acid (C16:0), palmitoleic acid (C16:1n-7), oleic acid (C18:1n-9), vaccenic acid (C18:1n-7), linoleic acid (C18:2n-6), ALA (C18:3n-3), arachidonic acid (C20:4n-6), EPA (20:5n-3), DPA (C22:5n-3), and DHA (C22:6n-3) in the FSO and FSO+TMT groups. TMT did not affect the amount of individual or total n-3 or n-6 fatty acids compared to Ctrl. On the other hand, only DPA and DHA were elevated in the hippocampi from FSO-treated TMT-intoxicated animals (FSO+TMT), which led to an increase in the total amount of PUFAs compared to TMT alone. No changes in n-6 fatty acids in the FSO+TMT group were observed. Consequently, the lowered n-6/n-3 ratio in the FSO+TMT group compared to the TMT group that we observed has a protective effect on the fatty acid profile of the hippocampus.

### 3.6. ALA Attenuated TMT-Induced Inflammatory Astrocyte Phenotype In Vitro

To discern the cell-specific beneficial actions of ALA, we have used the primary astrocyte culture to evaluate whether ALA might prevent TMT-induced prevailing pro-inflammatory astrocytes. The MTT analysis showed that the most appropriate dosage of ALA was 50 µM (testing 10, 50, 100, and 250 µM), which did not reduce the total mitochondrial activity of astrocyte cultures compared to control, untreated astrocytes. The vehicle control group is described in the Supplementary Material (Figure S1). Similarly, using the MTT assessment of the cultures’ metabolic rate, 5 µM of TMT was the chosen dosage for further experiments (tested concentrations 1, 2.5, 5, and 10 µM), since it was the lowest TMT concentration that consistently decreased the total mitochondrial activity by ~10% compared to the control group.

**Figure 6:**
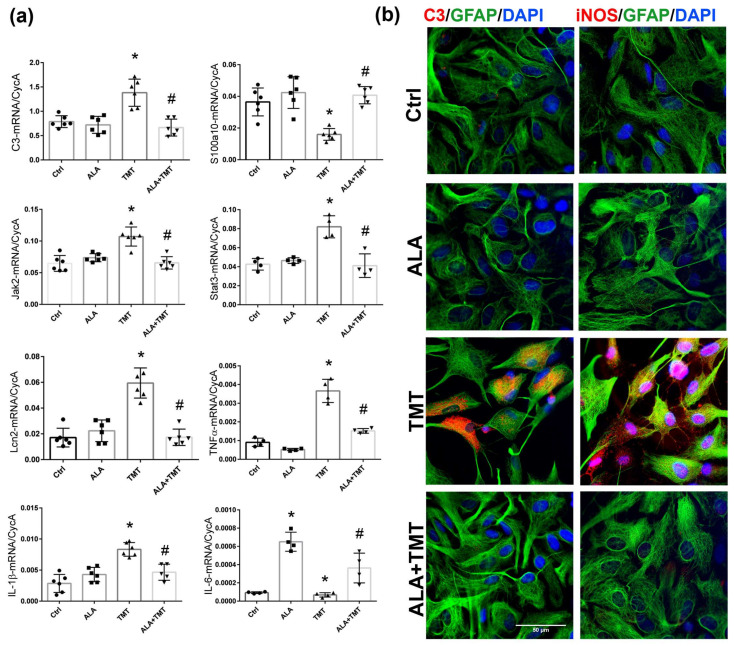
ALA prevented the expression of pro-inflammatory cytokines in cortical astrocytes in vitro. (**a**) The abundance of transcript coding C3, S100a10, Jak2, Stat3, Lcn2, TNFα, IL-1β, and IL-6 in the astrocytes in vitro. Bars represent mean target mRNA abundance relative to CycA (±SD) from *n* = 5 individual experiments. Significance shown inside the graphs: * *p* < 0.05 or less relative to Ctrl; # *p* < 0.05 or less relative to TMT. (**b**) Representative double immunofluorescence labeling directed to C3 (red)/GFAP (green) and iNOS (red)/GFAP (green) in control astrocytes (Ctrl), astrocytes pretreated with 50 μM α-linolenic acid (ALA), astrocytes treated with 5 μM trimethyltin (TMT) for 24 h, and astrocytes treated with 50 μM ALA+5 µM TMT (ALA+TMT). Cell nuclei were counterstained with DAPI (blue). Scale bar: 50 μm.

First, we performed a gene expression analysis of the neurotoxic marker C3; inflammatory mediators, such as IL-1β, lipocalin 2 (Lcn2), and TNF-α, which signify a prevailing A1-like proinflammatory phenotype; as well as the s100a10, STAT3, and JAK2 transcription factors, which signify a prevailing protective A2-like phenotype [16]. The results of two-way ANOVA for qPCR analysis are presented in the Supplementary Material, in Table S6. As already shown, a several-fold induction of all tested inflammatory genes has been observed 24 h after exposure to TMT (Figure 6a–i), implying the induction of pan-reactive astrocytes [12]. TMT-induced upregulation of C3-, IL-1β-, Lcn2-, Jak2-, and Stat3-mRNA (*p* < 0.001 for all examined genes relative to Ctrl) was fully prevented with ALA treatment (*p* < 0.001 for C3, *p* < 0.01 for IL-1β, *p* < 0.01 for Jak2, *p* < 0.001 for both Stat3 and Lcn2, compared to TMT; Figure 6). The prevailing astrocyte phenotype was also confirmed by showing that astrocytes exposed to TMT downregulated the expression of the S100a10 transcript (*p* < 0.01), while ALA prevented this downregulation (*p* < 0.001 compared to TMT group).

Cell morphology is another important aspect of astrocyte activation. Immunofluorescence labeling directed to GFAP and counterstaining with nuclear fluorescent stain DAPI have shown that after exposure to TMT, astrocytes reduced cell bodies, retracted cell processes, and increased intercellular space (Figure 6b). Immunofluorescence staining directed to C3 complement protein had shown that TMT induced massive expression of C3 in virtually all cells (Figure 6b). Pretreatment with ALA inhibited TMT-induced alterations in astrocyte cell morphology, as well as C3-ir. Since astrocytes produce NO in response to stressful stimuli for triggering neuroinflammatory mediators [52], we evaluated inducible nitric oxide synthase (iNOS)-ir, the enzyme which participates in NO synthesis. In unstimulated control cultures, GFAP-positive astrocytes were negative for iNOS (Figure 6b). After 24 h of stimulation with TMT, intensive labeling for iNOS was observed in most of the astrocytes, diffusely presented in the cytoplasm. ALA prevented TMT-induced iNOS-ir.

## 4. Discussion

Different dietary interventions, including fatty acids, can have a profound effect on the maintenance of health and the appearance of disease [17]. Since TMT intoxication can cause neurological syndromes and share common pathogenic pathways to neurodegenerative diseases such as AD, we used the pathological context of TMT to evaluate the potential of FSO as a preventive approach against the occurrence and progression of neurodegeneration and inflammation. In the current study, we observed that FSO given to animals before and over the course of TMT intoxication reduced hippocampal neuronal loss, prevented the activation of astrocytes, and inhibited their polarization toward the pro-inflammatory phenotype. These beneficial effects are accompanied by an increased intrahippocampal content of n-3 fatty acids.

As has been shown, the mechanism of neuronal death induced by TMT in rats has been linked essentially to apoptosis through the activation of the caspase8/caspase3 signaling pathway [46,53,54]. Similarly, we found increased expression of pro-apoptotic caspase3/Bax molecules accompanied by apparent morphological injury of pCA3 neurons at the early stage of TMT intoxication, as well as decreased expression of anti-apoptotic molecule Bcl-2. The application of FSO counteracted the effects of TMT, probably through the prevention of TMT-induced changes in the expression of apoptotic molecules and TMT-induced cell death in pCA3 as early as 7 days post intoxication, maintaining cell integrity and function. When massive neuronal loss was clearly noticed at the late stage of TMT intoxication, we could not detect changes in apoptotic molecules, except for a decrease in Bcl-2. However, the neuronal loss that was clearly noticed in this stage indicates that the mechanisms of neuronal cell death are still active, as observed by Gasparova and coworkers [55].

Many signaling pathways and/or signaling mediators, such as ERK, JNK, PI3K/Akt, and NF-κB p65 are activated in rodents following TMT [56,57,58]. In general, PI3K/Akt, ERK, and NF-κB signaling molecules are well known in regulating cell survival and cellular stress. We found that ERK was consistently activated at the late stage of TMT intoxication, which is in agreement with previous reports indicating that ERK is a key element of the neuroinflammatory pathway triggered by glial cells during the development of various neurodegenerative diseases [56,59]. Concomitantly, TMT suppressed p-Akt and its target NF-κB p65 subunit. Our data demonstrated that FSO prevented the overactivation of ERK, attenuated the decrease in p-Akt levels, and counteracted the observed alteration in the expression of the NF-κB p65 subunit, which exceeded control levels. It is known that activated NF-κB/p65 translocates to the nucleus, where it binds to and activates the transcription of the genes that are involved in neuroprotection [47]—one of them being BDNF, a major neuroprotective protein in the brain, the levels of which rise following various brain injuries [60,61], including TMT intoxication, where it is associated with the survival of immature progenitor neurons and will eventually lead to recovery [58]. In FSO-treated TMT-intoxicated hippocampi, BDNF-mRNA expression exceeded the increase observed after FSO and TMT. However, we detected discrepancies between the BDNF transcript and protein abundances. It is worth mentioning that BDNF-mRNA can be delivered in the form of silent mRNAs at synapses in extra-somatic compartments where they are locally translated in response to specific stimuli [62]; thus, discrepancies should not be unexpected. FSO+TMT increased mature BDNF protein abundance, but without changes in pro-BDNF expression. Whether the elevated translation of existing BDNF transcripts can help in the synthesis of pro-BDNF in order to preserve its basal levels has to be determined in some future experiments. Previous studies also showed that FSO induces an increase in BDNF levels following ischemic brain stroke, thus improving functional motor recovery [24]. Also, FSO supplementation can confer a number of health benefits in depressed women, including increased serum BDNF concentrations, along with improvements in depression status [22]. It is proposed that the release of mature BDNF into the extracellular milieu results in the rapid activation of TrkB receptors, NMDA receptors, and downstream signaling pathways to promote neuronal survival and enhance neurotransmission, stimulating neuroprotective mechanisms and leading to neurological improvement in different brain disease models [32].

Another factor that contributes to TMT-induced neurodegeneration is alterations in glutamatergic neurotransmission [7]. It was shown that concentrations of glutamate are significantly decreased at the late stage of TMT intoxication [63]. However, there are no data regarding possible TMT-induced alterations of NMDAR and glutamate transporters. Slight changes in the constitutive GluN1 and GluN2A subunits are probably not physiologically significant, but together with the decrease in glutamate transporter GLT-1 protein abundance, they might represent a compensatory mechanism for reduced glutamate concentration after TMT exposure. On the other hand, FSO treatment before and over the course of TMT-induced neurodegeneration slightly increased GluN1 protein abundance and preserved GluN2A at the control levels. It also decreased GluN2B protein levels, an NMDAR subunit which increases in abundance and/or whose activation may mediate severe neuronal injury [64]. Furthermore, PSD-95 is a critical synaptic protein that controls excitatory synaptic transmission and plasticity. It influences NMDA receptor transmission via the direct interaction and stabilization of specific NR2-containing NMDA receptors to the postsynaptic membrane. Together with cell loss, decreased PSD95 protein levels after TMT intoxication have been observed in brain tissue from AD subjects [65] and in models of AD [66]. Such downregulation of PSD-95 indicates a loss of synapses, which is prevented by FSO.

TMT-induced neuronal loss is associated with the activation of astrocytes. We previously showed that the activation of astrocytes was an early event following TMT exposure, triggered concomitantly with neuronal damage but before signs of cell death [10]. In our current study, as well as in our previous study [9], TMT induced massive astrogliosis in the hippocampal CA1 and DG. Elevated gene expression of C3 and s100a10, together with increased expression of main pro-inflammatory markers, may indicate that TMT-induced reactive astrogliosis exerts a complex molecular signature with a predominantly inflammatory phenotype [67] mainly localized around the sites of ongoing neurodegeneration [10]. It was found that astrocytes could express C3 and S100a10 simultaneously in a region-specific manner [68]. Also, it has been suggested that A1-like astrocytes release pro-inflammatory and neurotoxic factors, such as TNFα, IL-1β, nitric oxide, and reactive oxygen species, and lose their normal function of promoting neuronal survival [69]. Although astrocytic protective mechanisms are activated after TMT intoxication, we were interested in C3-positive astrocytes that expressed pro-inflammatory molecules and were located in the injured neuronal layers, which we observed previously [9]. Thus, we found that FSO prevented TMT-induced astrocyte reactivation and the expression of pro-inflammatory mediators, TNF-α, IL-1β, IL-10, IL-6, and a marker of harmful astrocytes, C3. FSO alone decreased the mRNA level of TNF-α; thus, it probably counteracts the whole inflammatory cascade and initiation of necroptosis [70]. This finding provided evidence that FSO protected rat brains against TMT intoxication partly via inflammatory regulation.

The neuroprotective/anti-inflammatory actions of FSO in TMT-induced neurodegeneration might be related to the observed increase in the intra-hippocampal content of omega-3 fatty acids such as ALA and its long-chain metabolites EPA, DPA, and DHA. Consequently, a lowered n-6/n-3 ratio has a protective effect on the fatty acid profile in the hippocampus [71]. The roles of DPA and DHA as neuroprotective agents are well studied to date [72,73]. On the other hand, ALA is a nutraceutical that targets multiple endogenous neuroprotective and neurorestorative pathways in numerous neurodegenerative disorders, which has been underestimated for many years [30,31,32,74]. Interestingly, ALA given as a pretreatment almost fully prevented CA1 neurodegeneration following ischemia or kainic acid-induced epileptic seizure [75]. Similarities between ALA-induced neuroprotective pathways parallel pathways activated with ischemia and other well-established chemical preconditioners, implying that ALA may be studied as a new natural preconditioner of the brain. ALA was shown to increase levels of BDNF, a widely distributed protein that carries out diverse functions in the brain, including neuronal maintenance, learning, memory, and neuronal survival [32]. Other proteins, such as HSP70, a heat shock protein, which acts as a protein chaperone, also have roles in regulating cell death [30], attenuating oxidative stress, and neuroinflammation [76]. Accordingly, we may assume that the increased intrahippocampal content of omega-3 fatty acids, ALA in particular, following FSO administration may induce tolerance in the hippocampal tissue and thus prevent TMT-induced neurodegeneration and inflammation. However, this assumption needs further clarification; since FSO was administered before and over the course of TMT, it is not possible at this moment to determine if the primary beneficial effect was preventative or therapeutic.

Given that ALA has been characterized as nutraceutical that can induce tolerance in the brain to adverse conditions, and given that it is increased following FSO administration, we wanted to examine its cell-specific effects and whether it could prevent the TMT-induced activation of astrocytes, which are the first responders to TMT-induced intoxication [10]. Our in vitro study showed that ALA pretreatment prevented TMT-induced polarization of cultured astrocytes towards the pro-inflammatory spectrum. Astrocytes did not acquire a reactive phenotype, characterized by a condensed cell body, retracted processes, and the upregulation of transcription factors and inflammatory mediators. Namely, ALA inhibited the upregulation of the mRNA levels of TNF-α, IL-1β, Lcn2, and C3, as well as the JAK/STAT cascade, which controls the expression of GFAP and thus contributes to activation of both pro-inflammatory and protective astrocytes [15,16]. At the same time, ALA prevented alteration in the protective S100a10 molecule, maintaining the protective astrocyte phenotype. The mechanisms underlying the anti-inflammatory effect of ALA might also involve interference with NO-producing enzymes such as iNOS [33]. However, we have to emphasize that ALA may be converted to DHA in astrocytes in vitro. Thus, the observed anti-inflammatory effects in vitro might be, in part, a result of DHA’s actions. This phenomenon awaits further research.

In conclusion, current findings indicate that FSO treatment given before and over the course of TMT neurodegeneration can alleviate cell loss, and inflammatory astrocytic activation probably increases the intrahippocampal content of omega-3 fatty acids. These data indicate the importance of a diet rich in polyunsaturated fatty acids for the prevention of the progression of diseases, raising the need for the investigation of the specific mechanisms of omega-3 fatty acid actions.

## Figures and Tables

**Table 1:** Fatty acid composition of FSO.

Fatty Acids	%
Saturated fatty acids (SFA)	
C16:0	7.96 ± 0.52
C18:0	4.29 ± 0.26
ΣSFA	12.25 ± 0.77
Monounsaturated fatty acids (MUFA)	
C16:1	0.18 ± 0.04
C18:1(n-9)	22.52 ± 0.88
C18:1(n-7)	0.00 ± 0.00
ΣMUFA	22.71 ± 0.84
Polyunsaturated fatty acids (PUFA)	
C18:3(n-6)	0.32 ± 0.09
C18:3(n-3)	39.16 ± 1.32
C20:3	0.22 ± 0.08
C20:4	0.00 ± 0.00
C20:5	0.00 ± 0.00
C22:4	0.00 ± 0.00
C22:5	0.00 ± 0.00
C22:6	0.00 ± 0.00
C18:2	25.34 ± 3.10
ΣPUFA	65.04 ± 1.61

**Table 2:** Primer sequences used for RT-qPCR.

Target Gene	Sequence (5′– –3′)
CycA (*Ppia*)	CAAAGTTCCAAAGACAGCAGAAAA CCACCCTGGCACATGAAT
Caspase-3 (*Casp3*)	GATGTCGATGCAGCTAACC TGTCTCAATACCGCAGTCC
Bax (*Bax*)	TGCTACAGGGTTTCATCCAG CCAGTTCATCGCCAATTCG
Bcl-2 (*Bcl2*)	TGGAAAGCGTAGACAAGGAGATGC CAAGGCTCTAGGTGGTCATTCAGG
TNF-α (*Tnf*)	CCCCCATTACTCTGACCCCT CCCAGAGCCACAATTCCCTT
IL-10 (*Il10*)	GCTCAGCACTGCTATGTTGC GTCTGGCTGACTGGGAAGTG
IL-6 (*Il6*)	CCGGAGAGGAGACTTCACAG ACAGTGCATCATCGCTGTTC
IL-1β (*Il1b*)	CACCTCTCAAGCAGAGCACAG GGGTTCCATGGTGAAGTCAAC
BDNF (*Bdnf*)	GGGACCAGGAGCGTGACAA GTCCGTGGACGTTTGCTTCT
C3 (*C3*)	GCGGTACTACCAGACCATCG CTTCTGGCACGACCTTCAGT
S100a10 (*S100a10*)	GTACCCACACCTTGATGCGT CGAAAGCTCCTCTGTCATTGG
Lcn2 (*Lcn2*)	GGATCAGAACATTCGTTCCA ATGGCAAACTGGTCGTAGTC
NF-kB (*Rela*)	AGCATGTACAGATTCTGGGGAG AGAGCCGACTATCGTACAGGG
Jak2 (*Jak2*)	TGGATCAAATCCGGGACAGT TCTTGAGCAGACAGCATCACAT
Stat3 (*Stat3*)	TGTGACACCAACGACCTGC ACACTCCGAGGTCAGATCCA

**Table 3:** List of primary antibodies.

Antibody Specificity	Source, Host Species	Dilution (in TBST or PBST)
Anti-Bax	Santa Cruz Biotechnology (Dallas, Texas, USA), mouse monoclonal	1:500
Anti-Bcl-2	Santa Cruz Biotechnology (USA), rabbit polyclonal	1:500
Anti-Casp3	Santa Cruz Biotechnology (USA), rabbit polyclonal	1:500
Anti-phospho-SAPK/JNK (Thr183/Tyr185)	Cell Signaling Technology (Danvers, Massachusetts, USA), rabbit polyclonal	1:500
Anti-JNK	Santa Cruz Biotechnology (USA), rabbit polyclonal	1:500
Anti-phospho-Akt (Ser473)	Cell Signaling Technology (USA), rabbit polyclonal	1:1000
Anti-total Akt	Cell Signaling Technology (USA), rabbit polyclonal	1:1000
Anti-phospho-p44/42 MAPK (Erk1/2) (Thr202/Tyr204)	Cell Signaling Technology (USA), rabbit polyclonal	1:1000
Anti-p44/42 MAPK (Erk1/2)	Cell Signaling Technology (USA), rabbit polyclonal	1:1000
Anti- NF-kB p65	Cell Signaling Technology (USA), rabbit polyclonal	1:2000
Anti-BDNF	Santa Cruz Biotechnology (USA), rabbit polyclonal	1:500
Anti- NMDA Receptor 1 (GluN1)	Cell Signaling Technology (USA), rabbit monoclonal	1:1000
Anti- NMDA Receptor 2A (GluN2A)	Merck Millipore (Burlington, Massachusetts, USA), rabbit monoclonal	1:1000
Anti-NMDA Receptor 2B	Abcam (Cambridge, UK), mouse polyclonal	1:4000
Anti-EAAT2 (GLT-1)	Abcam (UK), rabbit monoclonal	1:1000
Anti-PSD95, clone 7E3-1B8	Merck Millipore (USA), mouse monoclonal	1:2000
Anti-β-actin	Abcam (UK), mouse monoclonal- conjugated to HRP	1:10,000
Anti-β-tubulin	Santa Cruz Biotechnology (USA), mouse monoclonal	1:1000

**Table 4:** Fatty acids analysis of the hippocampus.

Hippocampal Fatty Acid Content (%)				
Fatty Acids	Ctrl	FSO	TMT	FSO+TMT
C16:0	5.72 ± 0.96	9.26 ± 2.25 *	7.83 ± 4.48	5.66 ± 0.98 ^a^
C16:1	0.23 ± 0	0.18 ± 0.04 *	0.29 ± 0.03 *	0.17 ± 0.02 ^b^
C18:0	29.14 ± 0.66	29.61 ± 1.68	29.19 ± 3.84	28.86 ± 0.55
C18:1(n-9)	23.89 ± 0.75	20.12 ± 1.34 *	23.41 ± 1.27	24.11 ± 0.83 ^a^
C18:1(n-7)	4.64 ± 0.17	6.43 ± 0.33 *	4.65 ± 0.41	4.54 ± 0.15 ^a^
C18:2	2.08 ± 1.06	0.84 ± 0.03 *	1.53 ± 0.25	1.62 ± 0.76
C18:3(n-6)	0.61 ± 0.04	0.6 ± 0.06	0.65 ± 0.06	0.6 ± 0.09
C18:3(n-3)	0.08 ± 0	0.14 ± 0.07 *	0.09 ± 0.02	0.09 ± 0.01
C20:3	1.1 ± 0.11	0.97 ± 0.13	1.17 ± 0.15	1.11 ± 0.15
C20:4	15.2 ± 0.61	13.63 ± 0.23 *	14.63 ± 0.99	14.99 ± 1.04 ^a^
C20:5	0.08 ± 0.01	0.06 ± 0.01 *	0.09 ± 0.02	0.1 ± 0.01
C22:4	2.29 ± 0.35	3.07 ± 1.92	2.31 ± 0.31	2.22 ± 0.47
C22:5	0.16 ± 0.02	0.2 ± 0.02 *	0.17 ± 0.02	0.25 ± 0.03 *^b^
C22:6	14.79 ± 0.39	14.9 ± 1.10	14 ± 1.31	15.59 ± 0.71 *^b^
ΣSFA	34.86 ± 1.63	38.87 ± 3.93 *	37.01 ± 8.32	34.52 ± 1.07 ^a^
ΣMUFA	28.76 ± 0.92	26.72 ± 1.70 *	28.35 ± 1.71	28.92 ± 0.95 ^a^
ΣPUFA	36.38 ± 2.6	34.4 ± 3.57	34.64 ± 3.13	36.57 ± 0.54 ^b^
Σn-6	21.27 ± 2.17	19.1 ± 2.38 *	20.28 ± 1.77	20.54 ± 0.55
Σn-3	15.11 ± 0.43	15.3 ± 1.19	14.36 ± 1.36	16.03 ± 0.68 ^b^
n-6/n-3	1.41 ± 5.07	1.25 ± 2.00 *	1.41 ± 1.3	1.28 ± 0.08 ^b^

Values are presented as mean ± SD. Different symbols denote values that are significantly different from the control group (Ctrl): * significantly different from Ctrl (*p* < 0.05 or less); ^a^ significantly different from the flaxseed oil (FSO) group (*p* < 0.05 or less); ^b^ significantly different from the trimethyltin (TMT) group (*p* < 0.05 or less); SFA, saturated fatty acids; MUFA, monounsaturated fatty acids; PUFA, polyunsaturated fatty acids.

## Data Availability

The data that support the findings of this study are available from the corresponding author upon reasonable request.

## Supplementary Materials

The following supporting information can be downloaded at: https://www.mdpi.com/article/10.3390/cells13141184/s1, Figure S1: Impact of α-linolenic acid (ALA) and trimethyltin (TMT) on metabolic activity of astrocytes. Bars represent Mean % ± SD from 5 separate culture preparations per group. Significance inside graph: * *p* < 0.05 compared to control group (Ctrl); Table S1: Results of Two-way ANOVA for mRNA and protein expression of components of cell death pathways in vivo; Table S2: Results of Two-way ANOVA for signaling pathways and BDNF expression in vivo; Table S3: Results of Two-way ANOVA for components of glutamatergic transmission in vivo; Table S4: Results of Two-way ANOVA for markers of astrogliosis and neuroinflammation in vivo; Table S5: Results of Two-way ANOVA for alterations of hippocampal FA composition; Table S6: Results of Two-way ANOVA for inflammatory mediators in primary astrocyte culture.
