# Supplementary material for: Flaxseed Oil Alleviates Trimethyltin-Induced Cell Injury and Inhibits the Pro-Inflammatory Activation of Astrocytes in the Hippocampus of Female Rats
Source: Cells. 2024 Jul 11;13(14):1184. doi: 10.3390/cells13141184 (PMC11274492; doi:10.3390/cells13141184)
Supplement: Supplementary file 1 [file cells-13-01184-s001.zip › cells-3055757-supplementary.pdf]

Supplementary material

Table S1. Results of Two-way ANOVA for mRNA and protein expression of components of cell death pathways *in vivo*

Examined molecules	Cell death pathways 7 and 21 dpi<br>Two-way ANOVA Results		
	FSO	TMT	FSO x TMT
**Bax 7 dpi**	F(1, 15) = 160.9; P < 0.001	F(1, 15) = 135.7; P < 0.001	F(1, 16) = 19.69; P < 0.01
**Casp3 7 dpi**	F(1, 15) = 6.094; P < 0.05	F(1, 15) = 18.56; P < 0.001	F(1, 15) = 8.40; P < 0.05
**Bcl-2 7 dpi**	F(1, 16) = 9.717; P < 0.05	F(1, 16) = 2.234; P = 0.1544; **NS**	F(1, 16) = 19.69; P < 0.01
**Bax 21 dpi**	F (1, 16) = 2.706 P = 0.1195; **NS**	F (1, 16) = 0.8679 P = 0.3654; **NS**	F (1, 16) = 36.54 P < 0.0001
**Bcl-2 21 dpi**	F (1, 17) = 0.01613 P = 0.9004; **NS**	F (1, 17) = 0.4933 P = 0.4920; **NS**	F (1, 17) = 2.147 P = 0.1611; **NS**
**Casp3 21dpi**	F (1, 16) = 5.477 P = 0.0326	F (1, 16) = 2.226 P = 0.1551; **NS**	F (1, 16) = 1.973 P = 0.1792; **NS**

NS, not significant; dpi, days post intoxication

Table S2. Results of Two-way ANOVA for signaling pathways and BDNF expression *in vivo*

Examined molecules	Signal molecules and BDNF 21 dpi<br>Two-way ANOVA Results		
	FSO	TMT	FSO x TMT
**p-Akt**	F (1, 16) = 22.57 P = 0.0002	F (1, 16) = 4.796 P = 0.0437	F (1, 16) = 1.617 P = 0.2216; **NS**
**p-ERK**	F (1, 20) = 40.41 P < 0.0001	F (1, 20) = 54.86 P < 0.0001	F (1, 20) = 3.563 P = 0.0737; **NS**
**NF-kB p65**	F (1, 16) = 20.77 P = 0.0003	F (1, 16) = 0.1069 P = 0.7479; **NS**	F (1, 16) = 18.88 P = 0.0005
**p-JNK 21dpi**	F (1, 20) = 1.841 P = 0.1900; **NS**	F (1, 20) = 0.05065 P = 0.8242; **NS**	F (1, 20) = 3.917 P = 0.0617; **NS**
**BDNF mRNA**	F (1, 14) = 46.84, P < 0.0001	F (1, 14) = 5.502, P = 0.0342	F (1, 14) = 2.107, P = 0.1687
**32 kDa BDNF**	F (1, 16) = 8.030 P = 0.0120; **NS**	F (1, 16) = 2.262 P = 0.1520	F (1, 16) = 0.004320 P = 0.9484; **NS**
**28 kDa BDNF**	F (1, 16) = 9.330 P = 0.0076	F (1, 16) = 19.15 P = 0.0005	F (1, 16) = 3.074 P = 0.0987; **NS**
**14 kDa BDNF**	F (1, 16) = 5.348 P = 0.0344	F (1, 16) = 14.91 P = 0.0014	F (1, 16) = 8.818 P = 0.0090

NS, not significant; dpi, days post intoxication

Table S3. **Results of Two-way ANOVA for components of glutamatergic transmission *in vivo***

Examined molecules	Components of glutamatergic transmission 21 dpi Two-way ANOVA Results		
	FSO	TMT	FSO x TMT
**GluN1**	F (1, 16) = 51.38 P < 0.0001	F (1, 16) = 9.234 P = 0.0078	F (1, 16) = 5.457 P = 0.0328
**GluN2A**	F (1, 20) = 0.04696 P = 0.8306; **NS**	F (1, 20) = 7.671 P = 0.0118	F (1, 20) = 4.360 P = 0.0498
**GluN2B**	F (1, 19) = 17.52 P = 0.0005	F (1, 19) = 0.3069 P = 0.5860; **NS**	F (1, 19) = 1.232 P = 0.2809; **NS**
**GLT-1**	F (1, 19) = 52.41 P < 0.0001	F (1, 19) = 1.282 P = 0.2716; **NS**	F (1, 19) = 11.26 P = 0.0033
**PSD95**	F (1, 19) = 30.75 P < 0.0001	F (1, 19) = 0.8142 P = 0.3782; **NS**	F (1, 19) = 17.89 P = 0.0005

NS, not significant; dpi, days post intoxication

Table S4. Results of Two-way ANOVA for markers of astrogliosis and neuroinflammation *in vivo*

Examined molecules	Markers of astrogliosis and neuroinflammation 21 dpi Two-way ANOVA Results		
	FSO	TMT	FSO x TMT
**GFAP**	F(1,15) = 61.21; P < 0.0001	F(1,15) = 87.49; P < 0.0001	F(1,15) = 142.8; P < 0.0001
**C3**	F(1,14) = 46.49; P < 0.0001	F(1,14) = 56.26; P < 0.0001	F(1,14) = 23.46; P = 0.0003
**S100a10**	F(1,15) = 8.328; P = 0.0113	F(1,15) = 6.011; P = 0.00269	F(1,15) = 3.699; P = 0.0736; **NS**
**TNFα**	F(1, 15) = 114.9, p < 0.0001	F(1, 15) = 12.45; P = 0.0030	F(1, 15) = 12.70, P = 0.0028
**IL-10**	F(1,16) = 21.17, p = 0.0003	F(1,16) = 28.784, P < 0.0001	F(1,16) = 4.391, P = 0.0524; **NS**
**IL-6**	F(1,16) = 51.05, p < 0.0001	F(1,16) = 26.17, P = 0.0001	F(1,16) = 28.92, P < 0.0001
**IL-1 β**	F(1,20) = 47.16, p < 0.0001	F(1, 20) = 67.87, P < 0.0001	F(1,20) = 58.36, P < 0.0001

NS, not significant

Table S5. Results of Two-way ANOVA for alterations of hippocampal FA composition

Examined FA	Hippocampal fatty acid composition Two-way ANOVA Results		
	FSO	TMT	FSO x TMT
**C16:0**	F (1, 18) = 6.855, P = 0.0174	F (1, 18) = 5.921, P = 0.0256	F (1, 18) = 10.49, P = 0.0046
**C16:1**	F (1, 18) = 102.1, P < 0.0001	F (1, 18) = 8.641, P = 0.0088	F (1, 18) = 19.44, P = 0.0003
**C18:0**	F (1, 18) = 2.993, P = 0.1008; **NS**	F (1, 18) = 3.991, P = 0.0611; **NS**	F (1, 18) = 0.5460, P = 0.4695; **NS**
**C18:1n-9**	F (1, 18) = 20.21, P = 0.0003	F (1, 18) = 19.62, P = 0.0003	F (1, 18) = 23.61, P = 0.0001
**C18:1n-7**	F (1, 18) = 46.67, P < 0.0001	F (1, 18) = 61.18, P < 0.0001	F (1, 18) = 55.85, P < 0.0001
**C18:2n-6**	F (1, 18) = 8.095, P = 0.0107	F (1, 18) =0.00861, P = 0.9271; **NS**	F (1, 18) = 3.651, P = 0.0721; **NS**
**C18:3n-6**	F (1, 18) = 4.865, P = 0.0406	F (1, 18) =0.02879, P = 0.8672; **NS**	F (1, 18) = 3.208, P = 0.0901; **NS**

**C18:3n-3**	F (1, 20) = 14.67 P = 0.0010; NS	F (1, 20) = 9.738 P = 0.0054	F (1, 20) = 11.59 P = 0.0028; NS
**C20:3n-6**	F (1, 18) = 10.66, P = 0.0043	F (1, 18) = 5.339, P = 0.0329	F (1, 18) =0.061, P = 0.8068; **NS**
**C20:4n-6**	F (1, 18) = 5.543, P = 0.0301	F (1, 18) = 8.996, P = 0.0077	F (1, 18) = 17.40, P = 0.0006
**C20:5n-3**	F (1, 18) = 2.746, P = 0.1148; **NS**	F (1, 18) = 28.51, P < 0.0001	F (1, 18) = 2.746, P = 0.1148; **NS**
**C22:4n-6**	F (1, 18) = 0.3424, P = 0.5657; **NS**	F (1, 18) = 1.619, P = 0.2195; **NS**	F (1, 18) = 2.649, P = 0.1210; **NS**
**C22:5n-3**	F (1, 18) = 51.20, P < 0.0001	F (1, 18) = 15.49, P = 0.0010	F (1, 18) = 6.272, P = 0.0221
**C22:6n-3**	F (1, 18) = 11.68, P = 0.0031	F (1, 18) = 0.1150, P = 0.7385; **NS**	F (1, 18) = 9.496, P = 0.0064
**ΣPUFA**	F (1, 18) =0.05198, P = 0.8222; **NS**	F (1, 18) = 0.3910, P = 0.5396; **NS**	F (1, 18) = 5.988, P = 0.0249
**Σn-6**	F (1, 18) = 5.636, P = 0.0289	F (1, 18) = 0.9681, P = 0.3382; **NS**	F (1, 18) = 5.432, P = 0.0316
**Σn-3**	F (1, 18) = 10.00, P = 0.0054	F (1, 18) = 0.2519, P = 0.6218; **NS**	F (1, 18) = 6.781, P = 0.0179
**n-6/n-3**	F (1, 18) = 20.74, p = 0.0002	F (1, 18) = 1.576, p = 0.2255; **NS**	F (1, 18) =0.01158, p = 0.9155; **NS**

NS, not significant

Table S6. Results of Two-way ANOVA for inflammatory mediators in primary astrocyte culture

**Inflammatory mediators *in vitro*<br>Two-way ANOVA Results**			
**Examined molecules**	**FSO**	**TMT**	**FSO x TMT**
**C3**	F(1, 16) = 17.38; p = 0.0007	F(1, 16) = 9.778; p = 0.0065	F(1, 16) = 10.01; p = 0.0060
**IL-1β**	F(1, 14) = 7.369; p = 0.0168	F(1, 14) = 15.44; p = 0.0015	F(1, 14) = 15.01; p = 0.00017
**Lcn2**	F(1, 20) = 27.13; p < 0.0001	F(1, 20) = 27.83; p < 0.0001	F(1, 20) = 44.92; p < 0.0001
**TNFα**	F (1, 12) = 39.97 P < 0.0001	F (1, 12) = 8.231 P = 0.0141	F (1, 12) = 4.808 P = 0.0488
**S100a10**	F(1, 16) = 17.50; p = 0.0007	F(1, 16) = 7.594; p = 0.00141	F(1, 16) = 9.693; p = 0.0067
**Jak2**	F(1, 12) = 6.833; p = 0.0226	F(1, 12) = 24.39; p = 0.0019	F(1, 12) = 18.82; p = 0.0003
**Stat3**	F(1, 12) = 16.01; p = 0.0018	F(1, 12) = 13.36; p = 0.0031	F(1, 12) = 23.03; p = 0.0004

NS, not significant

MTT Test

The effects of the alpha-linolenic acid (ALA), and trimethyltin (TMT) on the metabolic activity of astrocytes was assessed using the MTT (3-[4,5-dimethylthiazol-2-yl]-2,5-diphenyltetrazolium bromide assay. The MTT analysis showed that the most appropriate dosage of ALA was 50 µM (testing 10, 50, 100 and 250 µM) which have not reduced the total mitochondrial activity of astrocyte cultures compared to control, untreated astrocytes (Figure 1). Vehicle (0.2% ethanol) did not affect cell viability (data not shown). Similarly, using the MTT assessment of cultures metabolic rate, 5 µM of TMT was a chosen dosage for further experiments (tested concentrations 1, 2.5, 5 and 10 µM), since it was the lowest TMT concentration that consistently decreased the total mitochondrial activity for ~10% compared to the control group (Figure 1).

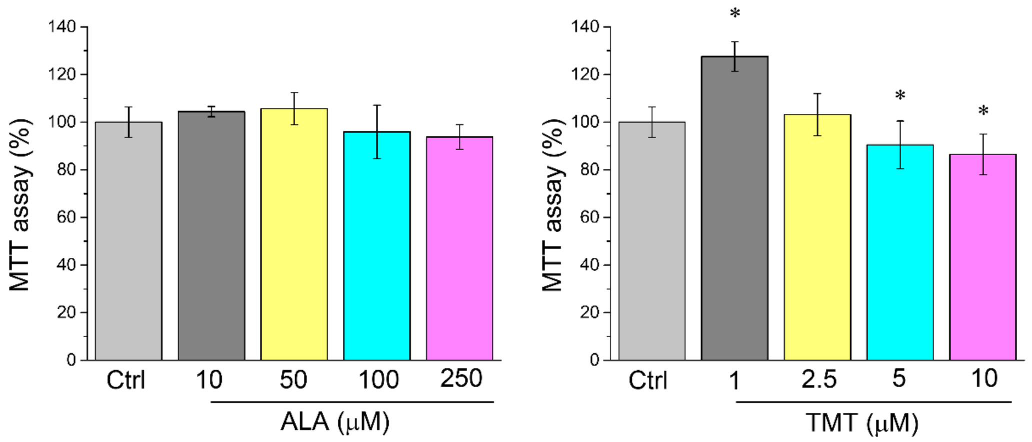

Figure S1. Impact of α-linolenic acid (ALA) and trimethyltin (TMT) on metabolic activity of astrocytes. Bars represent Mean % ± SD from 5 separate culture preparations per group. Significance inside graph: *$p < 0.05$ compared to control group (Ctrl).
